# Trend of viral load during the first, second, and third wave of COVID-19 in the Indian Himalayan region: an observational study of the Uttarakhand state

**DOI:** 10.3389/fmicb.2023.1279632

**Published:** 2024-01-15

**Authors:** Shailender Negi, Diksha, Deepjyoti Kalita, Neeraj Ranakoti, Ashish Negi, Diksha Kandwal, Shailesh Kumar Gupta, Yogendra Pratap Mathuria

**Affiliations:** ^1^Viral Research and Diagnostic Laboratory (VRDL), All India Institute of Medical Sciences, Rishikesh, India; ^2^Department of Microbiology, All India Institute of Medical Sciences, Guwahati, India; ^3^Department of Microbiology, All India Institute of Medical Sciences, Rishikesh, India

**Keywords:** COVID-19, SARS-CoV-2, cycle threshold, viral load, trend

## Abstract

India had faced three waves throughout the Coronavirus disease 2019 (COVID-19) pandemic, which had already impacted economic lives and affected the healthcare setting and infrastructure. The widespread impacts have inspired researchers to look for clinical indicators of severe acute respiratory syndrome coronavirus 2 (SARS-CoV-2) infection prognosis. Cyclic threshold values have been used to correlate the viral load in COVID-19 patients and for viral transmission. In light of this correlation, a retrospective study was conducted to assess the trend of viral load in clinical and demographic profiles across the three waves. Data of a total of 11,125 COVID-19-positive patients were obtained, which had a Ct value of <35. We stratified Ct values as follows: under 25 (high viral load), 25–30 (moderate viral load), and over 30 (low viral load). We found a significantly high proportion of patients with high viral load during the second wave. A significantly high viral load across the symptomatic and vaccinated populations was found in all three waves, whereas a significantly high viral load across age groups was found only in the first wave. With the widespread availability of real-time PCR and the limited use of genomic surveillance, the Ct value and viral load could be a suitable tool for population-level monitoring and forecasting.

## 1 Introduction

Coronavirus disease 2019 (COVID-19) is one of the deadliest infectious diseases caused by severe acute respiratory syndrome coronavirus 2 (SARS-CoV-2) that has affected millions of lives worldwide (Seok et al., 2019). The WHO declared the novel coronavirus outbreak a public health emergency of international concern (PHEIC) on 30 January 2020 and a pandemic on 11 March 2020, raising it to its highest level of alarm. As of 5 July 2023, a total of 0.76 billion COVID-19 cases and 6.9 million deaths have been reported worldwide (WHO Coronavirus (COVID-19), 2023). India has reported 44 million total cases of COVID-19 with half a million deaths (WHO Coronavirus Disease, 2023). Moreover, India has witnessed three massive waves: the first wave began in March 2020 and lasted till November 2020, the second wave ranged from March 2021 to May 2021, and the third wave ranged from January 2022 to March 2022 (Zirpe et al., 2021; Jayadevan et al., 2022). Among all three waves, the second wave appeared to be more deadly, with limited availability of vital therapies along with escalating cases and deaths accompanied by a shortage of hospital beds and oxygen (Asrani et al., 2021). The vast effects of the COVID-19 pandemic on healthcare systems have inspired researchers to look for clinical indicators of SARS-CoV-2 infection prognosis (Martínez et al., 2022). Cycle threshold (Ct) values in real-time reverse transcription–polymerase chain reaction (rRT-PCR) have been used to quantify the amount required for the target viral gene to pass the threshold and are inversely correlated with viral load (Tom and Mina, 2020). Several investigations have revealed a strong association between COVID-19 severity and disease progression and lower Ct values, which indicate increased viral load (Rao et al., 2020). The most common targets for RT-PCR coronavirus detection are conserved or highly expressed genes, such as structural spike glycoprotein (S) and nucleocapsid protein (N) genes, as well as non-structural RdRp and replicase open reading frame (ORF) 1ab genes (Cui et al., 2019). A correlation between viral load and Ct value was also assessed in influenza and respiratory syncytial (RSV) virus, but a significant relationship was observed only in the RSV (Reina et al., 2018). Similarly, the Ct value of SARS-CoV-2 was used to evaluate the viral load in the adult and elderly populations and to determine the role of these populations in virus transmission (Mishra et al., 2022). In view of this concept, the present study aimed to assess the viral load across the demographic and clinical profiles during the first, second, and third waves in India.

## 2 Materials and methods

### 2.1 Study design and study site

The present study used a retrospective cross-sectional design for evaluating Ct values of SARS-CoV-2-positive samples tested in the Viral Research and Diagnostic Laboratory (VRDL), Department of Microbiology, AIIMS, Rishikesh, during the first, second, and third waves.

### 2.2 Data source

We used the ICMR COVID-19 data portal for extracting SARS-CoV-2-positive data during the first, second, and third waves. The Ct values of the SARS-CoV-2-positive samples were obtained from the RT-PCR in the BIO-RAD CFX 96TM system (Bio-Rad Laboratories, Inc., USA) using COVIDsure Multiplex Realtime RT-PCR Kit (Trivitron Healthcare Pvt. Ltd.). The viral RNA isolation from nasopharyngeal/oropharyngeal swabs was performed by an automated nucleic acid extraction system (KingFisher™ Flex system) (Thermo Fisher Scientific, USA) using MagMAX Viral/Pathogen Nucleic Acid Isolation Kit (Thermo Fisher Scientific, USA).

The VRDL, AIIMS, Rishikesh, has been authorized by the ICMR for the testing and reporting of SARS-CoV-2 samples in the ICMR COVID-19 data portal. The extracted data had a unique identifier for each sample, SRF number, generated at the time of sample collection and must be required during reporting. In addition, the data comprised demographic, clinical, hospitalization, vaccination, and reporting parameters. In the present study, we stratified the Ct values as follows: under 25 (high viral load), 25–30 (moderate viral load), and over 30 (low viral load). In addition, we categorized age into three groups: under 18 years (young), 18–60 years (adult), and over 60 years (elderly).

### 2.3 Study sample and sample size

Samples tested positive (Ct < 35) for SARS-CoV-2 and had at least one Ct value reported among the two confirmatory genes, ORF or RdRp, from various districts of Uttarakhand and Uttar Pradesh during the three waves were included in the study. The mean Ct value was obtained if both genes were reported. A total of 11,125 patients were found eligible for the study.

### 2.4 Statistical analysis

Statistical analyses were performed using Microsoft Excel, R (The R foundation), and GraphPad Prism. Descriptive analyses were carried out to determine the patient characteristics across the different COVID waves and were presented using frequency and proportion. The median and interquartile range were reported for Ct value after testing for normality using the Shapiro–Wilk test. The statistical comparison of the Ct values and viral load among patients’ demographic, hospitalization, and clinical and vaccination statuses were made using the Mann–Whitney U tests, Kruskal–Wallis test, chi-square tests, or Fisher exact tests, as appropriate. The confidence interval was set at 95% and the significance level at 5%.

## 3 Results

### 3.1 Patient characteristics across the first, second, and third waves of the COVID-19 pandemic

In total, we gathered data from 11,125 COVID-positive patients, of which 6,030 suffered during the first wave, whereas 3,398 and 1,697 suffered during the second and third waves, respectively. In all three waves, adults were an overwhelming majority (78–87%), and the women-to-men ratio was approximately 2:3. There was a rapid increase in the community cases (59 to 97%), whereas the hospitalization rate declined (41 to 3%) with the subsequent wave. Overall, few patients had received vaccination (3.48%) and, approximately, three of five patients were asymptomatic (Table 1).

**Table 1:** Patient characteristics in the first (March–November, 2020), second (March–May, 2021), and third (January–March, 2022) waves of the COVID-19 pandemic.

Characteristics	Wave 1 (*N* = 6,030)	Wave 2 (*N* = 3,398)	Wave 3 (*N* = 1,697)
	n (%)	n (%)	n (%)
Age groups			
≤18 years (young)	339 (5.6)	183 (5.4)	131 (7.7)
19–60 years (adult)	4,711 (78.1)	2,885 (84.9)	1,474 (86.9)
>60 years (elderly)	980 (16.3)	330 (9.7)	92 (5.4)
Sex			
Male	4,227 (70.1)	2089 (61.5)	1,011 (59.6)
Female	1803 (29.9)	1,309 (38.5)	686 (40.4)
Patient status			
Community	3,555 (59)	3,260 (96.3)	1,647 (97.1)
Hospitalized	2,469 (41)	126 (3.7)	50 (2.9)
Clinical status			
Asymptomatic	4,074 (67.6)	2,213 (65.1)	1,219 (71.8)
Symptomatic	1956 (32.4)	1,185 (34.9)	478 (28.2)
Vaccination status			
Non-vaccinated	6,030 (100)	3,062 (90.1)	1,646 (97)
Vaccinated	0 (0)	336 (9.9)	51 (3)
All values rounded up to 1 decimal			

### 3.2 The trend of viral load across the three waves of the COVID-19 pandemic

**Figure 1:**
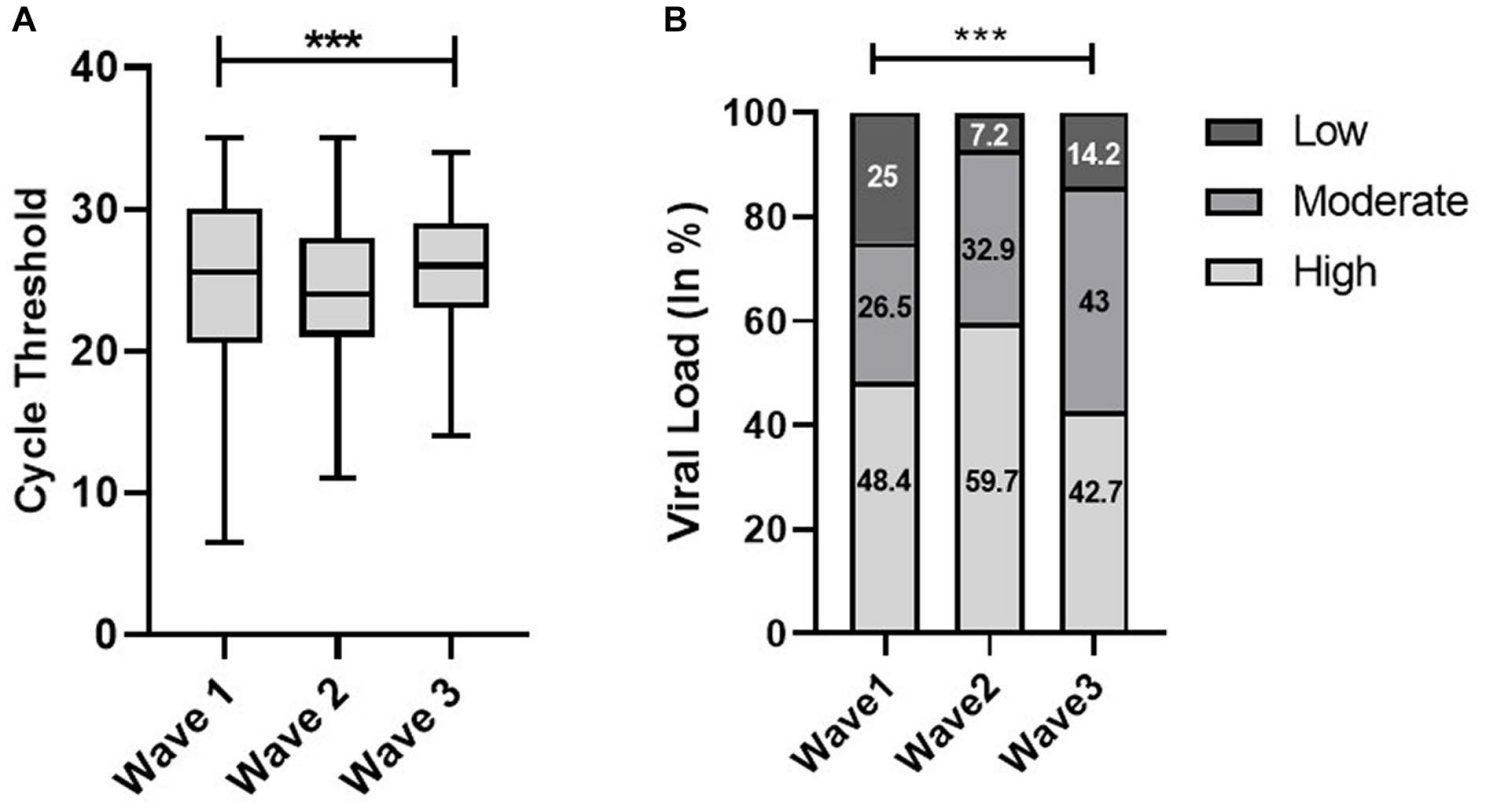
Distribution of cycle threshold (Ct) values **(A)** and viral load **(B)** across the first, second, and third waves of the COVID-19-positive cases: midlines indicate the median, boxes indicate interquartile ranges, and whiskers indicate the upper and lower adjacent values (within 1.5 times the interquartile range).

The Shapiro–Wilk test of normality determined the skewness of Ct values. The median Ct value in the second wave was significantly low, 24 (interquartile range [IQR], 7) as compared to the first and third waves, which was 26 (IQR, 10) and 26 (IQR, 6), respectively. In terms of viral load, we observed a significant trend that increased from the first wave to the second wave and then decreased in the third wave (Figure 1). The Indian SARS-CoV-2 Genomics Consortium (INSACOG) data also revealed the circulation of a more severe Delta variant (B.1.617) of SARS-CoV-2 in the second wave compared to B.1.1.529 (Omicron variant) in the third wave (INSACOG, 2023).

### 3.3 Distribution of viral load in different patient populations

**Figure 2:**
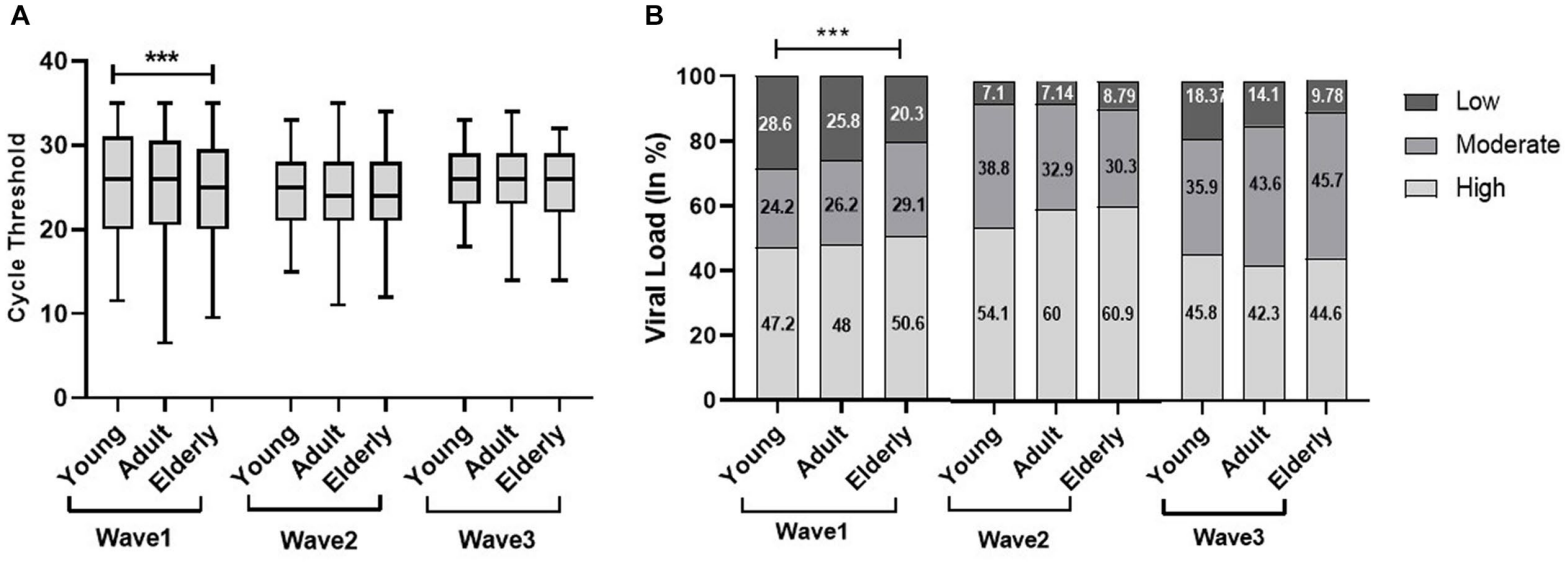
Age-specific comparisons of cycle threshold (Ct) values and viral load across the three COVID waves: midlines indicate the median, boxes indicate interquartile ranges, and whiskers indicate the upper and lower adjacent values (within 1.5 times the interquartile range).

**Figure 3:**
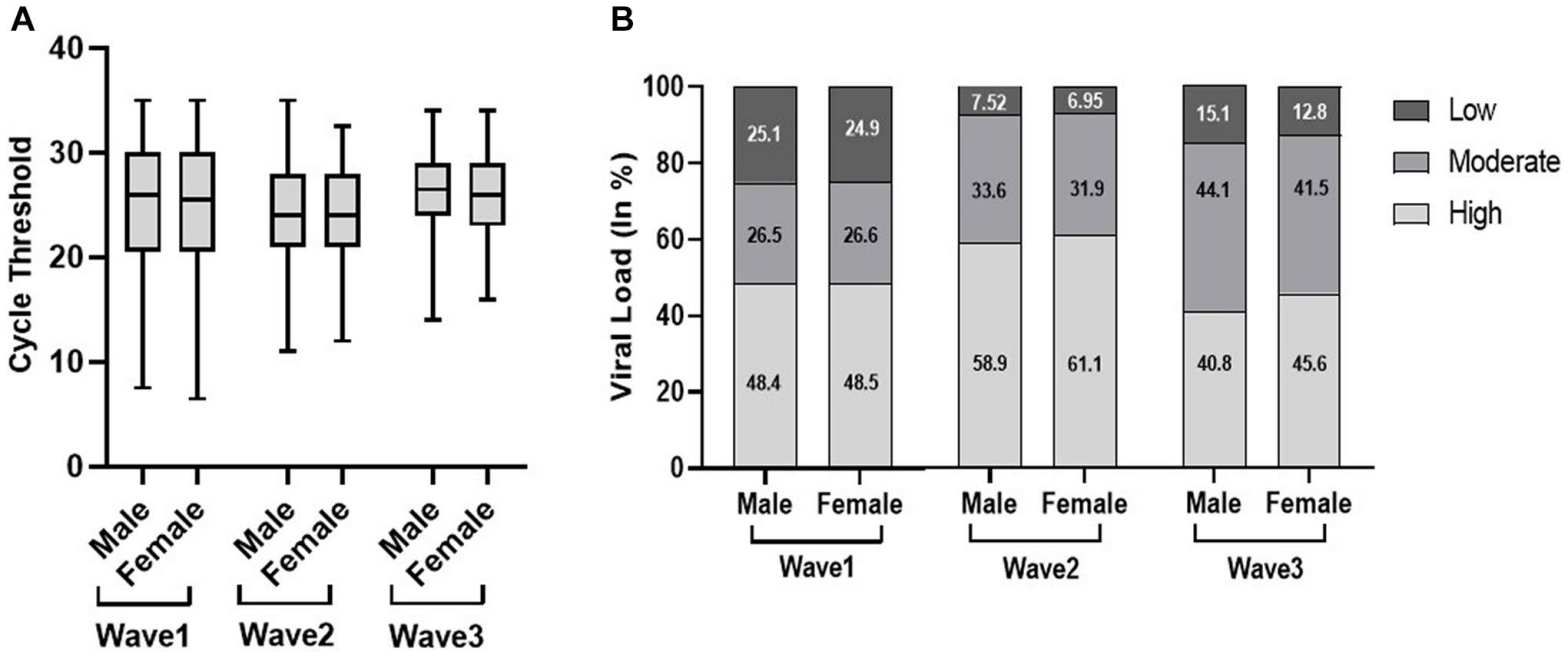
Distribution of cycle threshold (Ct) values **(A)** and viral load **(B)** in male and female patients across the three COVID waves: midlines indicate the median, boxes indicate interquartile ranges, and whiskers indicate the upper and lower adjacent values (within 1.5 times the interquartile range).

In the first wave, the elderly population had a significantly low median Ct value (25) as compared to the young (26) and adult population (26); however, there was no significant difference observed in the second and third waves. Similarly, the viral load data of the first wave indicated a significantly high viral load (50.6%) in the elderly population compared to adults (48%) and the young population (47.2%). Although there was no significant difference observed in viral load across the second and third waves, the young population in the third wave had a high viral load (45.8%) as compared to the elderly (44.6%) and adult populations (42.3%) (Figure 2). We did not find any statistically significant difference in the Ct value and viral load across sex, but the viral load among women is higher in all three waves (Figure 3).

**Figure 4:**
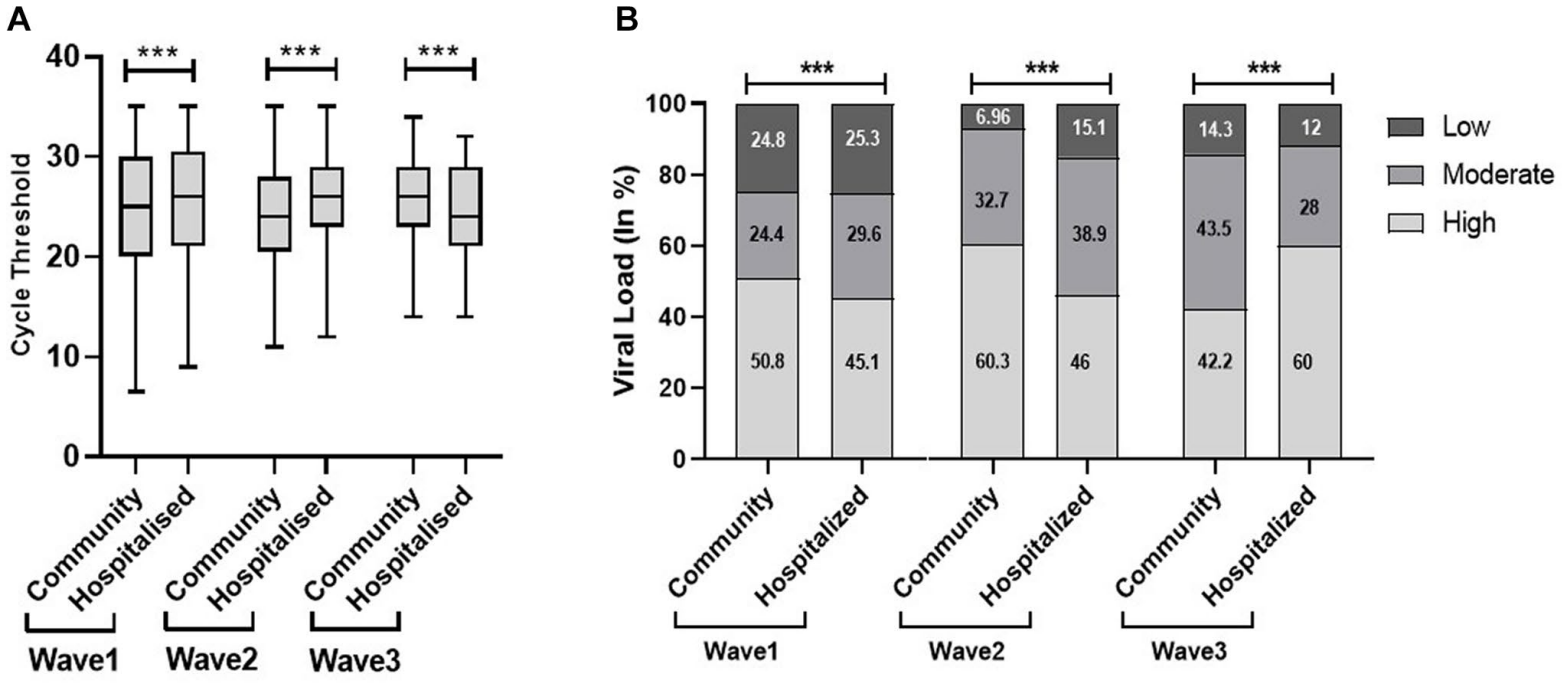
Cycle threshold (Ct) values **(A)** and viral load **(B)** of community and hospitalized patients across the three COVID waves: midlines indicate the median, boxes indicate interquartile ranges, and whiskers indicate the upper and lower adjacent values (within 1.5 times the interquartile range).

There was a significantly low median Ct value in the community population in the first and second waves; however, it was significantly low in the hospitalized patients in the third wave. The viral load data echo the Ct findings, as shown in Figure 4. The present study found out more hospital admissions even with low viral load in the first wave, indicating the severity as well as lack of preparedness during the first wave of the pandemic.

**Figure 5:**
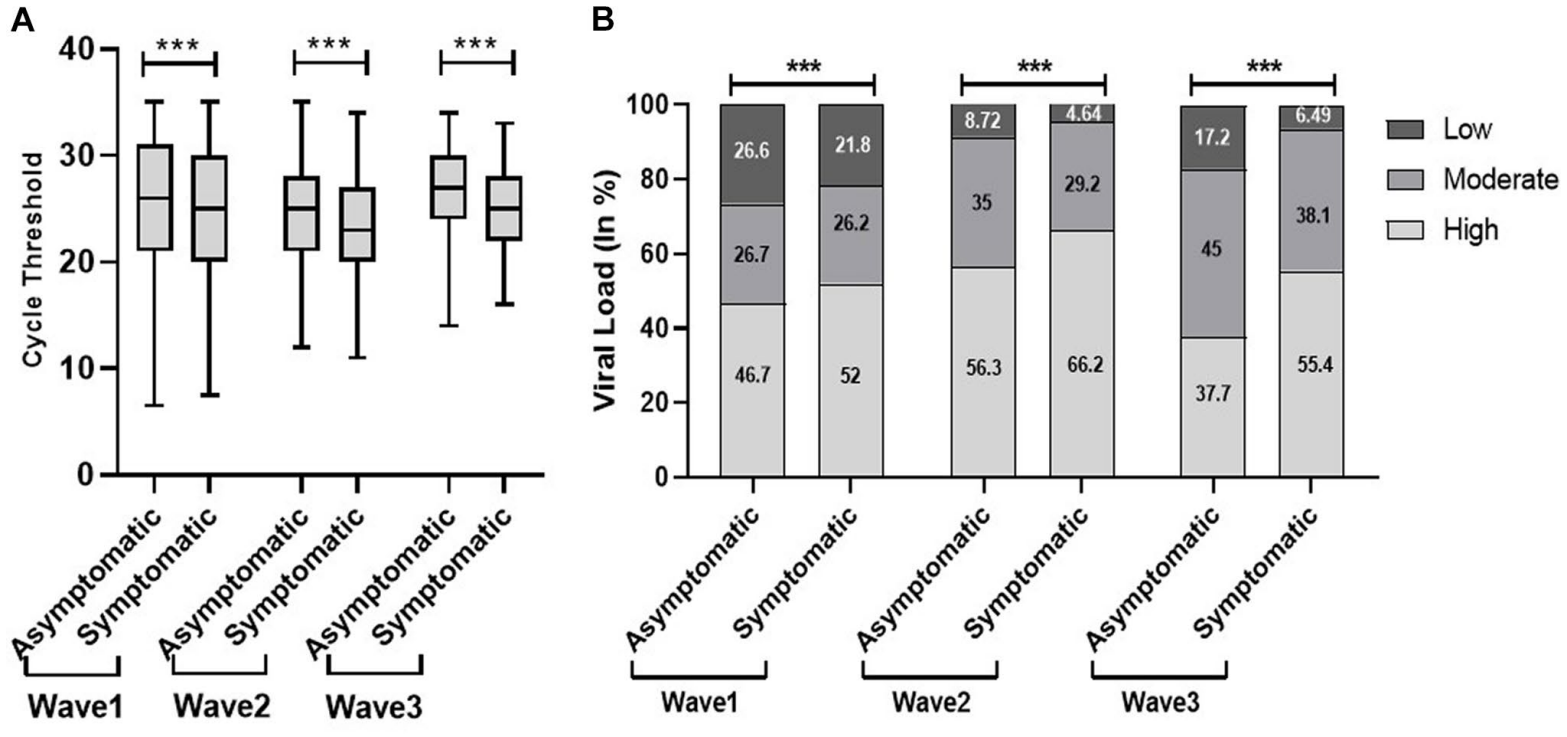
Comparison of cycle threshold (Ct) values **(A)** and viral load **(B)** among patients’ clinical status during the three COVID waves: midlines indicate the median, boxes indicate interquartile ranges, and whiskers indicate the upper and lower adjacent values (within 1.5 times the interquartile range).

**Figure 6:**
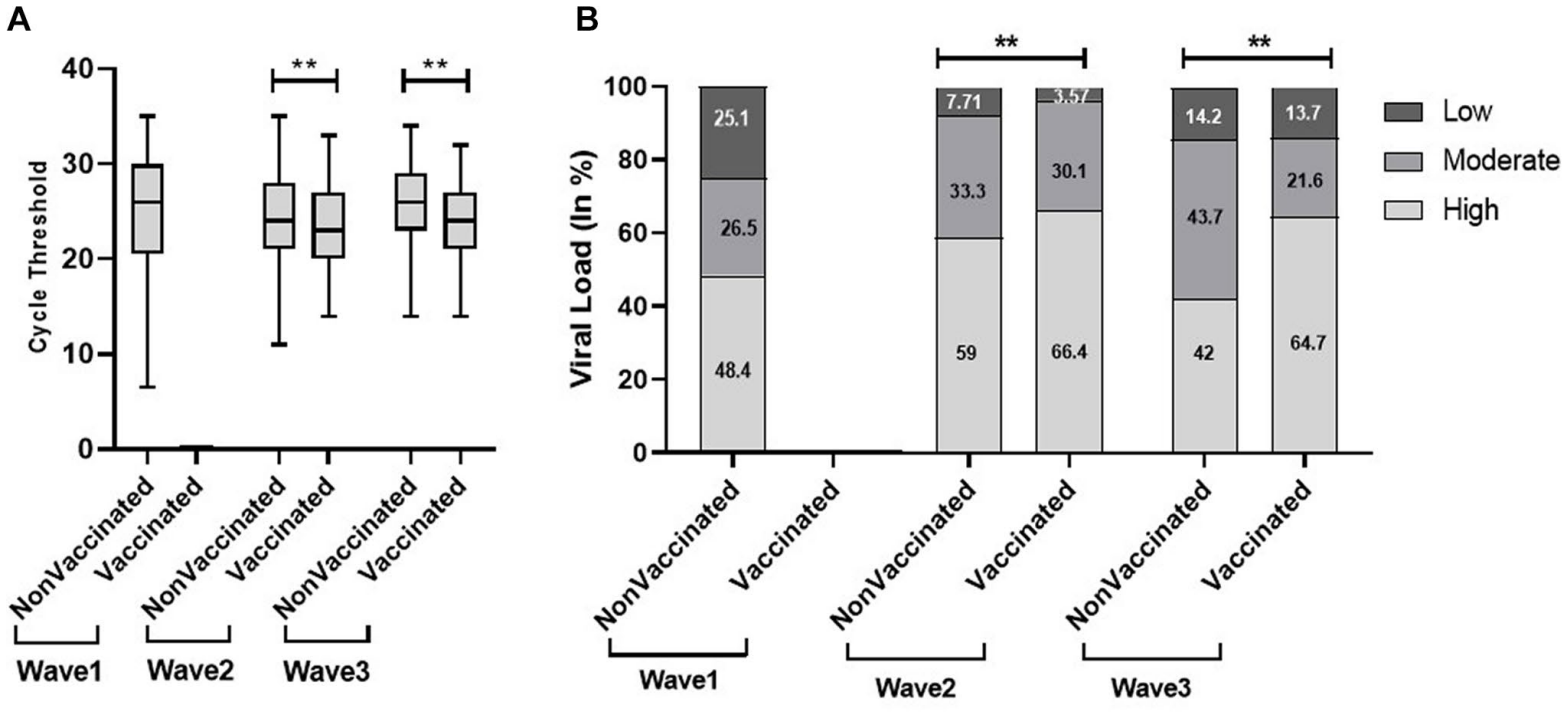
Comparison of cycle threshold (Ct) values **(A)** and viral load **(B)** in vaccinated and non-vaccinated patients across the three COVID waves: midlines indicate the median, boxes indicate interquartile ranges, and whiskers indicate the upper and lower adjacent values (within 1.5 times the interquartile range).

We found a significantly low median Ct value among the symptomatic population compared with the non-symptomatic population across the three waves (Figure 5). Similar results were obtained with viral load. The viral load data showed more symptomatic cases with low viral load during the first wave (21.8%) compared to the second (4.6%) and third (6.5%) waves. Our study found a higher proportion of vaccinated patients with significantly low Ct value and high viral load compared with non-vaccinated in the second and third waves. In addition, the lower proportion of the non-vaccinated population with high viral load in the third wave (42%) compared to the first (48.4%) and second (59%) waves indicated a decrease in the severity of the pandemic as well as the development of herd immunity (Figure 6).

## 4 Discussion

The best way to diagnose SARS-CoV-2 infection in the laboratory is the detection of viral RNA using a real-time reverse transcriptase PCR (rRT-PCR) assay from respiratory tract specimens. The single data points derived from real-time PCR amplification plots are called threshold cycles or Ct values. The Ct value has an inverse relationship with the amount of virus present in a particular sample. A low Ct value indicates a high level of genetic material, which is often associated with an increased level of infection (Rao et al., 2020). The Ct value is a semi-quantitative tool for the broad characterization of viral load as low, moderate, and high (Indian Journal of Medical Specialities, 2023). Ct value being a proxy of viral load can serve as an indicator for infectivity and severity of disease. Our study found a high proportion of the population with low Ct value and high viral load in the second wave compared to the first and third waves. In addition, Mishra et.al observed a high proportion of low Ct value in the second wave in positive samples (Mishra et al., 2022). In India, the sudden increase in cases in the second and third waves possibly indicates the unlocking of social activities as well as the transmission of a more infectious variant, B.1.617 (Delta variant) and B.1.1.529 (Omicron), respectively (Choudhary et al., 2021). The high transmissibility and infection rate of SARS-CoV-2 during the second wave led to viral spread in the asymptomatic population, especially those who stayed inside, contributing to an increase in positivity and death (Choudhary et al., 2021). Besides the high viral load in the second wave, we did not observe substantial hospitalization. Similar findings were observed in a comparative study from a tertiary care hospital in India, determining high mortality due to tachypnoea, hypoxia, and lack of an aggressive management system (ICU) could be the possible reason for the reduction in hospitalization percentage (Tendulkar et al., 2023).

Among various predictors of the severity and outcomes of COVID-19, age plays a significant role associated with an increased risk of the disease, leading to morbidity as well as mortality (Statsenko et al., 2022). A study by Hasanoglu et al. (2021) showed a statistically significant correlation between age and viral load. Few studies concluded the direct association between old age and high viral load and disease severity (Qian et al., 2020; To et al., 2020). Our findings revealed the severity of the first wave predominantly on the elderly population compared to the second and third waves. The possible reason could be age-related comorbidities, especially acute respiratory distress syndrome (ARDS) in the elderly population (Chen et al., 2020). The corresponding results obtained by Sun et al. demonstrate the catastrophic effects of COVID-19 in elderly patients compared to young patients, with a shorter average duration from the onset of symptoms to death. These findings suggested the quick advancement of the disease in older people than in younger ones (Sun et al., 2020). Despite the severity of the pandemic, the younger population had milder infections during the first and second waves. Good inflammatory response and delayed development of angiotensin-converting enzyme 2 led to better outcomes in children with COVID-19. However, concern had been raised primarily for non-vaccinated children during the third wave, and our findings revealed a high proportion of the young population with high viral load during the third wave (Sun et al., 2020).

The effect of the SARS-CoV-2 virus generates various clinical responses ranging from symptomatic to asymptomatic and more severe infections that require critical care (Gülbudak et al., 2021). According to a report, 40–45% of the SARS-CoV-2 cases do not exhibit any symptoms. Initially, there were different opinions regarding the infectiousness of asymptomatic patients for the COVID-19 spread. During the evaluation of viral load from different samples, Hasanoglu et al. demonstrated high viral load from nasopharyngeal/oropharyngeal samples of asymptomatic COVID-19 patients than symptomatic patients and considered these asymptomatic cases as an invisible part of the iceberg (Hasanoglu et al., 2021). In contrast, numerous studies, including the current study, demonstrated low Ct value and high viral load in symptomatic patients compared to asymptomatic patients (Gülbudak et al., 2021; Kociolek et al., 2021; Strutner et al., 2021). Despite these parameters, few researchers found no significant difference in Ct value among symptomatic and asymptomatic individuals (Lee et al., 2020; Louie et al., 2022). Therefore, there is a need for a longitudinal study for assessing the possible relationship between Ct value and symptoms.

One of the main reasons behind the successive waves, besides human influence, is the evolution of SARS-CoV-2. In India, the Delta and Omicron variants of SARS-CoV-2 were responsible for the second and third waves, respectively. It is a well-known fact that the Omicron variant has higher transmissibility and infectivity than Delta (ECDC) (European Centre for Disease Prevention and Control, 2023). Despite their higher transmissibility and infectivity, high mortality and less hospitalization had been observed during the second wave. The application of a low Ct value for the prediction of COVID-19 outcomes in hospitalized patients is considered a useful tool during the peak COVID wave (Kurzeder et al., 2022). One study compared low Ct value with poor disease outcomes in unvaccinated hospitalized patients, whereas another study utilized the Ct value (<26) as a risk score in predicting patient mortality along with other parameters in hospitalized patients of COVID-19 (Wright et al., 2021; Kurzeder et al., 2022). However, few studies limit its application to early warning indicators due to its marked variation in community patients, but one of the studies rejects the use of Ct value as a prognostic factor or a marker in community patients of COVID-19 (Martínez et al., 2022). The present study found a low Ct value among hospitalized COVID-19 patients during the third wave in our tertiary care hospital.

The best strategy to prevent from COVID-19 is the successful administration of vaccines as it minimizes diseases, acute illnesses, and deaths caused by the SARS-CoV-2. India started its COVID-19 vaccination program on 16 January 2021. Limited studies exploit the relationship of Ct value with the vaccination status of individuals. A study by Acharya et al. did not find any significant association between Ct value and vaccination status. Despite the vaccination program, we found a significantly high viral load in the vaccinated population compared with the non-vaccinated population in the second and third waves, which could be due to the fact that the newer strains most likely evade the immune response that was initially triggered by a vaccine using an ancestral wild-type virus. The recurrent mutations and evolution in newer strains help in viral evasion from neutralizing antibodies in vaccinated individuals. We also found a high viral load in the non-vaccinated population in the second wave, and the reason could be the severity of the Delta variant, whereas the decrease in viral load in the non-vaccinated population in the third wave could be possibly explained by less severity of the Omicron variant as well as the development of herd immunity within the community (Mandal et al., 2021). More scientific studies are required for a better understanding of the role of Ct value in vaccinated patients.

The paucity of data related to the severity of the COVID-19 infection, mainly the specific symptoms, and co-infections, was the major limitation of this study. Though some researchers observed no or minimal role of co-infections in COVID-19 severity, the topic is still debatable and demands further research (Gupta et al., 2023).

## 5 Conclusion

Our study highlights the trend of viral load across the three waves in India. The increase in the proportion of high viral load during the second wave indicated the severity of the wave; however, the reduction in high viral load proportion suggested the decline in severity of the third wave. With the development of highly effective vaccines and an increase in immunity against the infection, the pandemic had shown a downward trend over a period, thereby allowing countries to return to normal life, which has led the WHO to announce COVID-19 as a no longer public health emergency on 5 May 2023. Despite this, the world is still facing 1.5 lakh new cases and 497 deaths weekly. Although the Ct value can potentially be used to improve diagnosis, management, and control, it is highly denounced in clinical settings for specific patient management due to its dependency on various factors such as kits, instruments, target genes, or biological material. With the widespread availability of real-time PCR and the limited use of genomic surveillance for predicting pandemic surge in resource-constrained and heavily populated countries, including India, the Ct value or viral load could be a suitable indicator for the early diagnosis of COVID-19 as well as population-level monitoring of COVID-19 dynamics and forecasting in subsequent waves, which can help in restricting the transmission of the virus.

## Data availability statement

The raw data supporting the conclusions of this article will be made available by the authors, without undue reservation.

## Ethics statement

The studies involving humans were approved by Institutional Ethics Committee, AIIMS Rishikesh. The studies were conducted in accordance with the local legislation and institutional requirements. The human samples used in this study were acquired from a by- product of routine care or industry. Written informed consent for participation was not required from the participants or the participants' legal guardians/next of kin in accordance with the national legislation and institutional requirements.

## Author contributions

SN: Conceptualization, Data curation, Formal analysis, Investigation, Software, Writing – original draft, Writing – review & editing. Diksha: Visualization, Writing – original draft, Writing – review & editing. DeK: Formal analysis, Resources, Supervision, Writing – review & editing. NR: Data curation, Methodology, Writing – review & editing. AN: Data curation, Resources, Writing – original draft. DiK: Methodology, Writing – original draft. SG: Investigation, Supervision, Writing – original draft, Writing – review & editing. YM: Conceptualization, Data curation, Investigation, Project administration, Validation, Visualization, Writing – original draft, Writing – review & editing.
